# Alterations of Gut Microbiome and Fecal Fatty Acids in Patients With Polycystic Ovary Syndrome in Central China

**DOI:** 10.3389/fmicb.2022.911992

**Published:** 2022-07-01

**Authors:** Gailing Li, Zhenguo Liu, Fang Ren, Huirong Shi, Qian Zhao, Yi Song, Xunjie Fan, Xiaojun Ma, Guijun Qin

**Affiliations:** ^1^Gynecology Department, The First Affiliated Hospital of Zhengzhou University, Zhengzhou, China; ^2^Department of Infectious Diseases, The First Affiliated Hospital of Zhengzhou University, Zhengzhou, China; ^3^Endocrinology and Metabolic Diseases, The First Affiliated Hospital of Zhengzhou University, Zhengzhou, China

**Keywords:** polycystic ovary syndrome, gut microbiome, fecal fatty acids, 16S rRNA gene sequencing, untargeted metabolomics

## Abstract

**Objective:**

The purpose of this study was to elucidate the characteristics of the gut microbiome in patients with Polycystic ovary syndrome (PCOS) and analyze the alterations of fecal fatty acid metabolism, so as to further provide the pathogenesis of PCOS.

**Methods:**

Fecal samples from the PCOS group (*n* = 31) and healthy control group (*n* = 27) were analyzed by 16S rRNA gene sequencing and untargeted metabolomics. Peripheral venous blood was collected to measure serum inflammation and intestinal permeability. Finally, the correlation analysis of intestinal flora, fecal metabolites, and laboratory indicators was carried out.

**Results:**

Serum D-lactate content in the PCOS group was higher than that in the control group. There was no significant difference in microbial α diversity and β diversity between PCOS patients and healthy controls. Peptostreptococcaceae and Bacteroidales S24-7 group existed significant differences between PCOS patients and healthy controls. Based on linear discriminant analysis selection, 14 genera including *Klebsiella*, *Enterobacteriaceae*, and *Gammaproteobacteria* were dominant in patients with PCOS, while 4 genera, including *rumenococcus* (*Ruminocaccaceae UCG 013*), *prewortella* (*Prevotellaceae UCG 001*), and *erysipelas* (*Erysipelatoclostridium*), were dominant in healthy controls. Compared with PCOS with Body mass index (BMI) < 24, patients with BMI ≥ 24 have multiple dominant genera including *Abiotrophia* and *Peptostreptococcaceae*. Moreover, serum levels of free testosterone and androstenedione were positively correlated with *Megamonas*, while total testosterone was negatively correlated with *Alistipes*. Additionally, fecal contents of acetic acid and propionic acid in patients with PCOS were significantly higher than those in healthy controls. *Eubacterium_coprostanoligenes_group* and *Alistipes* were positively correlated with 6 kinds of fatty acids.

**Conclusion:**

Specific intestinal flora fecal fatty acids and serum metabolites may mediate the occurrence and development of PCOS. PCOS patients with different body sizes have specific intestinal flora.

## Introduction

Polycystic ovary syndrome (PCOS) is one of the most prevalent endocrine and metabolic diseases and the main cause of infertility in women of reproductive age, affecting approximately 8–13% of women worldwide (Rotterdam, 2004; Teede et al., 2019). PCOS may be a complex polygenic disease with strong epigenetic inheritance and influenced by factors including diet and lifestyle (Escobar-Morreale, 2018). It is characterized by a series of symptoms, such as hirsutism, alopecia, acne, and rare ovulation or non-ovulation, as well as a series of endocrine disorders, such as hyperandrogenism, insulin resistance, and hyperinsulinism. PCOS can have profound and long-term health consequences (Teede et al., 2010). Despite the development of PCOS treatment, including treatment for hyperandrogenism and insulin resistance, and ovulation induction therapy (Jayasena and Franks, 2014; Azziz et al., 2016), its prevention and long-term control are still not ideal, because the precise underlying pathogenesis and mechanisms for these endocrine and metabolic disturbances remain largely unclear (Azziz et al., 2016; Yang et al., 2021). In addition, bariatric surgery has been shown to be effective in severely obese patients with PCOS, but the benefit-to-risk ratio still needs to be carefully evaluated (Palomba et al., 2021). Thus, it is still urgent to explore its pathogenesis to provide evidence for treatment.

Gut microbiota plays a wide range of functions in humans, such as digestion of food, maturation of the host immune system, synthesis of short-chain fatty acids (SCFAs), vitamins, and amino acids, protection against pathogens, and regulation of bone mineral density, angiogenesis, fat metabolism, and drug metabolism (Macfarlane and Macfarlane, 1997; Ley et al., 2006; Nenci et al., 2007; Mitreva and Human Microbiome Project Consortium, 2012; Pickard, 2015). Previous studies have implicated that the alteration of gut microbiota may be involved in various diseases, including type 2 diabetes mellitus (T2DM), obesity, and nonalcoholic fatty liver disease (Canfora et al., 2019), and the incidence of these diseases also increased in PCOS (Azziz et al., 2016; Kumarendran et al., 2018; Zeng et al., 2021). Importantly, gut dysbacteriosis is found in clinical studies and animal research of PCOS (Lindheim et al., 2017; Liu et al., 2017; Kusamoto et al., 2021; Liang et al., 2021; Lin et al., 2021). Gut microbiota could be served as a key diagnostic biomarker in PCOS and cause insulin resistance (Yang et al., 2021). The possible mechanism is that gut permeability increased after gut dysbacteriosis, which causes lipopolysaccharide to enter the body’s circulation system, activate the immune system and inflammatory response, and promote insulin resistance of PCOS patients (Tremellen and Pearce, 2012; Duan et al., 2021). Moreover, the gut microbiota is one of the major regulators of circulating estrogen, contributing to the development of PCOS (Baker et al., 2017). Based on those, we speculate that gut dysbacteriosis is common in PCOS, and is closely related to the occurrence and development of PCOS.

Gut microbiota participates in the overall metabolism of the host, and metabolites play important roles in the microbial community. SCFAs are lipid metabolites of the gut microbiome, which are involved in gene expression, inflammation, differentiation, and apoptosis in host cells (Canfora et al., 2019). It has been found that short-chain fatty acid metabolic abnormalities caused by gut microbiological abnormalities are related to insulin resistance and hyperandrogenism in PCOS patients (Kumarendran et al., 2018; Zeng et al., 2022). Gut microbiota can affect the body’s insulin sensitivity through the inflammatory reaction mediated by SCFAs (Lindheim et al., 2017). It can be seen that the relationship between PCOS and SCFAs metabolism of gut microorganisms is also worthy of further exploration by the scientific community.

In this study, 16S rRNA sequencing and lipidomics were applied to the fecal samples of 58 participants to analyze the altered characteristics of gut microflora and short-chain fatty acid metabolism in PCOS patients. We observe changes in intestinal barrier function and inflammation in patients with PCOS. In addition, correlation analysis was performed on laboratory indicators, intestinal flora, and fecal fatty acids.

## Materials and Methods

### Study Profile

Through strict inclusion and exclusion criteria, the present study prospectively collected fecal and serum samples from 58 participants in central China, including 31 patients with PCOS and 27 healthy controls, for analysis. This study was reviewed and approved by the Ethics Committee of the First Affiliated Hospital of Zhengzhou University (2021-KY-0069). All selected participants understood the risks and benefits of the study and signed informed consent. Detailed inclusion, exclusion, and diagnostic criteria can be found in the Supplement Method. The gut microbial characteristics of 31 PCOS and 27 healthy controls were described and compared. According to body mass index (BMI), the included PCOS patients were divided into PCOSF group (BMI ≥24 kg/m2, *N* = 12) and PCOST group (BMI < 24 kg/m2, *N* = 19). The gut microbial characteristics of PCOS patients with different body types were analyzed and compared. In addition, association analysis was performed for laboratory indicators, intestinal microbiota, and SCFAs. We must evaluate intestinal barrier function and inflammatory response of PCOS and healthy controls.

### Fecal Samples Collected, DNA Extracted, and Concentration Determined

Each participant (non-menstrual) provided a fresh stool sample between 06:30 am and 08:30 am and collected about 1,000 mg of clean stool from the stool center with a sterile spoon. The samples were divided into sterile boxes of 200 mg each and refrigerated at −80°C for 30 min until used for testing. A fecal genomic DNA extraction kit (EZNA^®^ Soil DNA Kit) was used to collect total DNA from the feces of PCOS patients and healthy controls. The detailed steps of DNA extraction and purification were detailed in the Supplement Method.

The concentration of nucleic acid and organic matter was detected by using the Nanodrop2000 nucleic acid concentration analyzer. The absorbance value of nucleic acid (A260), the absorbance value of organic matter (A280), and the absorbance value of carbohydrate (A230) were obtained. When A260/A230 > 2.0 and A260/A280 ≈ 1.9 the purity of DNA extract was higher. TAE 1 × buffer solution was used to prepare 1% agarose gel, and the DNA extract was separated (105 V, 30 min). The size and completeness of the DNA bands were observed under the gel imager. If the bands were clean, with large molecular weight and single linearity, the extracted DNA was relatively complete.

### Gut Microorganism PCR Amplification, MiSeq Sequencing, MiSeq Library Construction, and Data Processing

The V3-V4 variable region sequence of bacterial 16S rRNA gene was used as the target, and 338F-806R with Barcode sequence was used as the primer for PCR amplification. The TransStartFastpfu DNA polymerase was used to amplify in ABI GeneAmp 9,700 PCR apparatus. Each sample was repeated three times. The PCR products of the same sample were mixed and detected by 2% agar gel electrophoresis. PCR products were recovered by gel-cut with AxyPreDNA gel recovery kit, eluted with Tris–HCl buffer, detected by 2% agarose gel electrophoresis, denatured with sodium hydroxide, and single-stranded DNA fragments were produced. The PCR products were quantified using the QuantifluorTM-ST blue fluorescence quantification system. Sequencing was performed on Illumina MiSeq 2^*^300 bp high-throughput sequencing platform. The detailed steps of MiSeq sequencing are described in the Supplement Method. The TruSeqTM DNA Sample Prep Kit was used to construct the MiSeq library. The raw Illumina read data for all samples were deposited in the European Bioinformatics Institute European Nucleotide Archive database (accession number: PRJNA823867).

### Bioinformatics Analysis

In this study, all sequencing data were analyzed using the Meiji Bio-Information Cloud Platform. By sampling OTUs analysis, the Shannon index calculated by R program package “Vegan” was used to measure and display the microbial diversity of the samples (Oksanen, 2011), while Chao index and ACE index were used to measure and display the microbial species number of the samples. Microbial community distribution and the degree of difference between each group of samples were shown by Principal coordinate analysis (PCOA), Principal component analysis (PCA), and Non-metric multi-dimensional scaling (NMDS) of the R package.^1^

Taxonomic analysis and comparison of two or more groups of bacteria, including Phylum, Genus, Class, Order, and Family, were performed by Wilcoxon rank-sum test and Kruskal–Wallis test. Based on the relative abundance of the standard matrix, we use the LEfSe method^2^ analysis between PCOS patients and healthy controls stool samples of microbial communities (Brown et al., 2011). The Kruskal-Wallis rank sum test (*p* < 0.05), and then Linear discriminant analysis (LDA) was used to evaluate the effect size of each feature [with LDA score (log10) = 2 or 2.5 as the cut-off value; Gill et al., 2006]. In addition, the Spearman correlation coefficient was used to analyze the correlation between gut flora and clinical indicators and SCFAs.

### Fatty Acid Analysis, GC–MS Analysis, and Data Analysis

The supernatant of human feces was collected for GC–MS analysis. Detailed treatment of fecal samples please see the Supplement Method. Agilent 7890B gas chromatography and Agilent 5977B mass spectrometry were used to detect the production of 11 metabolites in human feces, including acetic acid, propionic acid, isobutyric acid, butyric acid, iso-valeric acid, valeric acid, hexanoic acid, heptanoic acid, octanoic acid, nonanoic acid, and capric acid. Detailed detection procedures were described in the online supplement method. Agilent MassHunter Qualitative Analysis B.04.00 software was used to convert the raw data into a general format. In the R software platform, the XCMS package is used to identify material signal peaks, correct retention time, and carry out the integration (Smith et al., 2006). The standard curve is used for quantification and internal standard correction. The expression difference of metabolites in PCOS and healthy controls were analyzed.

### Blood Samples Collected, Inflammatory Indexes, and Intestinal Permeability Indexes Determined

Fasting blood samples from the participants were collected using procoagulant tubes after 8 to 12 h of fasting on the second to the fifth day of menstruation. After the blood samples were collected, the blood samples were left standing at room temperature for 30 min and centrifuged at 4,000 r/min for 10 min. The supernatant was transferred into 1.5 ml EP tubes and immediately stored in a −80°C refrigerator for later use.

Enzyme-linked immunosorbent assay (ELISA) was used to detect serum levels of IL-6, TNF-α, lipopolysaccharide-binding protein (LBP), and diamine oxidase (DAO) in PCOS patients and healthy women in the control group. Serum D-lactic acid (D-LA) levels in PCOS patients and healthy women in the control group were determined by colorimetry. Detailed detection methods and procedures refer to the online supplement method.

### Statistical Analysis

SPSS 20.0 was used for data statistics and analysis, and GraphPad Prism 7 was used to make various statistical graphs. The categorical and continuous variables were expressed as mean (standard deviation) or median (quartile range) and percentage, respectively. If they were in line with normal distribution and homogeneity of variance, an independent sample *T*-test was used for the comparison of quantitative data between groups. If the variance is not normal or homogenous, the rank-sum test is performed. The classified data were tested by the chi-square test. *p* < 0.05 (two-tailed) was the significance test level.

## Results

### Analysis of Basic Clinical Data Between PCOS and Healthy Controls

Age, height, weight, BMI, hirsuties, acne, waist circumference, hip circumference, total testosterone (TT), sex hormone binding globulin (SHBG), double hydrogen testosterone (DHT), free testosterone (FT), fasting insulin (FINS) and fasting blood glucose (FBG) were compared between PCOS patients and healthy controls. The results were shown in Table 1. Compared with healthy controls, the hairy symptoms and acne symptoms were more significant in PCOS patients (*p* < 0.001), and the serum levels of DHT (*p* < 0.05), FT (*p* < 0.01), TT, FINS, and FBG (*p* < 0.001) were significantly increased. While there were no significant differences in age, height, weight, BMI, waist circumference, hip circumference, and WHR between the two groups (*p* > 0.05).

**Table 1:** Analysis of basic clinical data of PCOS and healthy controls.

	Healthy controls (***n*** = 27)	PCOS (***n*** = 31)	Value of *p*
Age (year)	27.0 ± 4.90	24.35 ± 4.48	0.0562
Height (cm)	162.8 ± 4.5	159.1 ± 5.6	0.0580
Weight (kg)	63.25 ± 10.74	61.37 ± 11.45	0.5249
BMI (kg/m^2^)	23.89 ± 3.95	24.19 ± 3.87	0.7652
Ferriman-Gallway hair score/crinosity	1.00 ± 1.00	6.13 ± 2.77	<0.0001^*^
Acne score	0.59 ± 1.05	10.83 ± 8.30	<0.0001^*^
Waist Circumference (cm)	81.00 ± 11.75	82.18 ± 12.89	0.7190
Hip Circumference (cm)	98.04 ± 8.26	97.16 ± 7.85	0.6807
WHR	0.82 ± 0.08	0.84 ± 0.08	0.3423
TT (nmol/L)	0.37 ± 0.13	2.43 ± 10.13	0.02964^*^
SHBG (nmol/L)	41.06 ± 20.32	23.22 ± 13.88	0.0468^*^
DHT (pgl/ml)	12.35 ± 5.78	15.84 ± 7.17	0.0481^*^
FT (pg/ml)	2.09 ± 0.54	2.83 ± 1.02	0.0013^*^
FINS (mmol/L)	6.09 ± 1.73	10.20 ± 8.25	0.0204^*^
FBG (mmol/L)	5.14 ± 0.37	5.73 ± 0.71	0.0403^*^

The categorical and continuous variables were expressed as mean (standard deviation) or median (quartile range) and percentage, respectively. If they were in line with normal distribution and homogeneity of variance, an independent sample T-test was used for the comparison of quantitative data between groups. If the variance is not normal or homogenous, the rank-sum test is performed. The classified data were tested by the chi-square test. A *p* < 0.05 (two-tailed) was the significance test level. PCOS, Polycystic ovary syndrome, WHR, Waist hip rate, TT, total testosterone, SHBG, sex hormone binding globulin, DHT, double hydrogen testosterone, FT, free testosterone, FINS, fasting insulin, and FBG, fasting blood glucose. *p ≤ 0.05.

### Analysis of Serum Inflammatory Indexes and Gut Permeability Indexes Between PCOS and Healthy Controls

**Figure 1:**
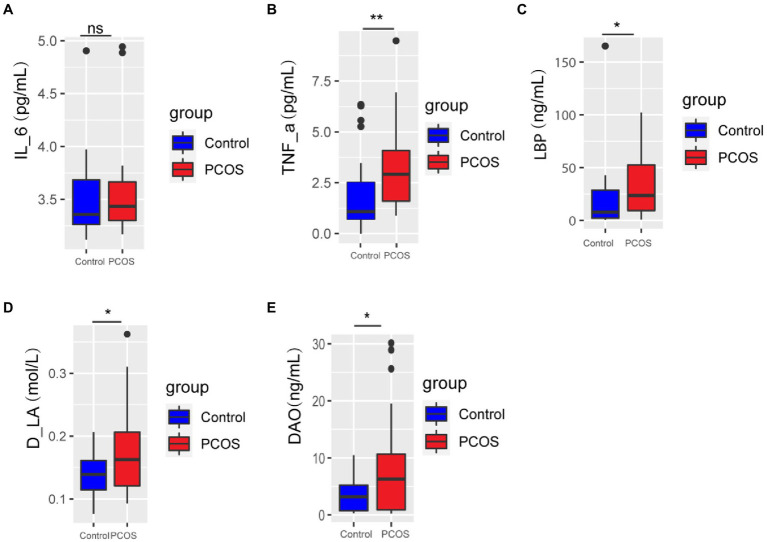
**(A-E)** Inflammation and gut permeability analysis of PCOS and healthy controls. A *p* < 0.05 (two-tailed) was the significance test level. PCOS, Polycystic ovary syndrome, LBP, Lipopolysaccharide-binding protein, D-LA, D-lactic acid, DAO, Diamine oxidase. ^*^p ≤ 0.05; ^**^p ≤ 0.01; and ^***^p ≤ 0.001. •outlier.

Serum levels of IL-6, TNF-α, Lipopolysaccharide-binding protein (LBP), D-lactic acid (D-LA), and Diamine oxidase (DAO) were compared between PCOS patients and healthy controls. As shown in Figure 1, serum levels of TNF-α, LBP, D-LA, and DAO in PCOS patients were significantly higher than those in healthy controls (*p* < 0.05), and there was no significant difference in serum IL-6 level between the two groups (*p* > 0.05). These results suggest that PCOS patients have impaired intestinal barrier function and a low degree of chronic inflammation in the body.

### Analysis of SCFAs in Feces of PCOS and Healthy Controls

**Figure 2:**
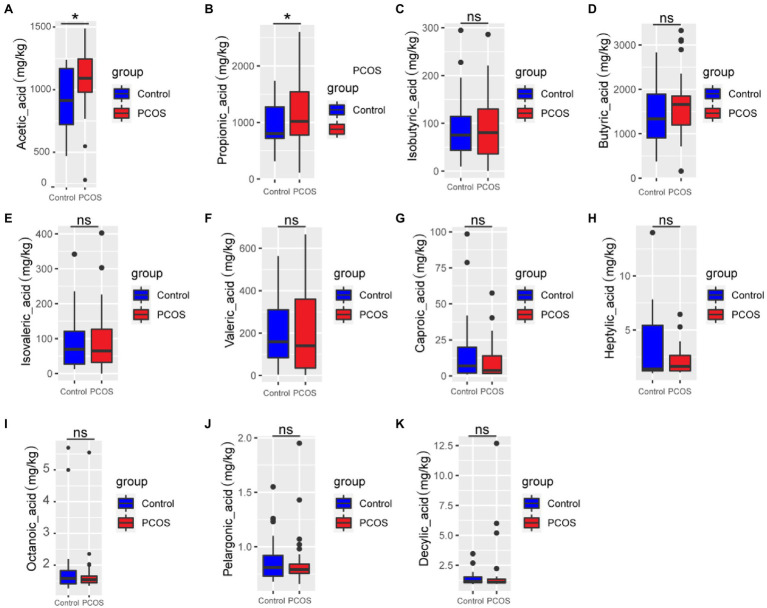
**(A-K)** Quantitative analysis of 11 SCFAs metabolites in samples from PCOS and healthy controls. A *p* < 0.05 (two-tailed) was the significance test level. PCOS, Polycystic ovary syndrome. ^*^p ≤ 0.05; ^**^p ≤ 0.01; and ^***^p ≤ 0.001. •outlier.

GC–MS was used to analyze 11 kinds of SCFAs in fecal samples from PCOS patients and healthy controls. The total ion effluent, retention time, and standard curves of the target compounds were shown in Supplementary Figure 1 and Supplementary Table 1, respectively. At the same time, quality control (QC) was carried out on the SCFAS test data, and the results were shown in the Supplementary Table 2. Mann–Whitney U method was used to further analyze the differences in SCFAs metabolism between the two groups. As shown in Figure 2, the fecal metabolism of acetic acid (*p* < 0.05) and propionic acid (*p* < 0.05) in PCOS was significantly higher than that of healthy controls.

### Analysis of Species Composition and Differences of Gut Flora Between PCOS and Healthy Controls

#### Gut Microflora Diversity Analysis of PCOS and Healthy Controls

**Figure 3:**
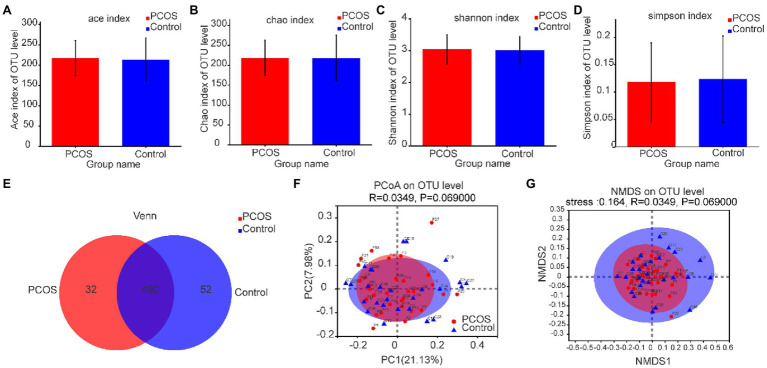
**(A–D)** Comparison of gut microbial α diversity index between PCOS and healthy controls. There was no significant difference in α diversity between the two groups. **(E)** A Venn diagram shows the number of unique and common OTUs between the two groups. **(F,G)** PCoA and NMDS based on OTU distribution showed no significant difference in β diversity between the two groups. PCOS: polycystic ovary syndrome (*N* = 31); Control: healthy controls (*N* = 27); PCoA, Principal coordinate analysis; NMDS, Non-metric multi-dimensional scaling; OTUs, Operational Taxonomic Units.

As shown in Figures 3A–D, according to the measurement and comparison of the diversity index ACE, Chao, Shannon Index, and Simpson Index, there was no significant difference in the abundance and uniformity between PCOS and healthy controls (*p* > 0.05).

Based on the distribution of gut microbial OTUs, PCoA and NMDS were used to display the spatial distribution of gut microbial community in PCOS and healthy controls. Venn diagram based on OTUs distribution was further used to display the microbiome distribution of PCOS and healthy controls. As shown in Figure 3E, the total abundance of OTUs in the two groups reached 574, of which 490 OTUs were shared by the two groups, but 32 OTUs still existed independently in PCOS. These isolated PCOS microbiome may be the key microbiome related to the development of PCOS. The results are shown in Figures 3F,G. The confidence ellipses of PCOS and healthy controls overlapped in a large area, indicating that the distribution of gut microbiome of the two groups was not significantly different. However, it should be noted that some microbiome still existed independently in PCOS or healthy controls.

#### Analysis and Comparison of Gut Microbiota in Phylum and Genus Between PCOS and Healthy Controls

To further analyze the phylogeny and degree of difference between PCOS and healthy controls, the Kruskal–Wallis rank-sum test was used to analyze and compare the differences in the abundance of gut microbiota between the two groups at the levels of Family and Genus.

**Figure 4:**
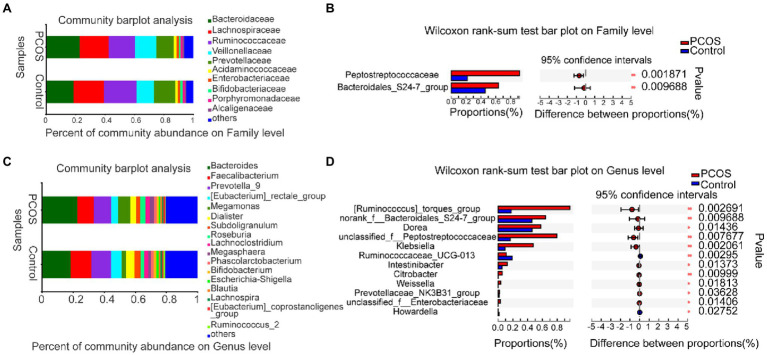
**(A,B)** At family level, the composition and differences of gut microbiota between PCOS and healthy controls were observed. **(C,D)** Composition and differences of gut microbiota at genus level between PCOS and healthy controls. The difference of intestinal flora between the two groups was compared by STAMP analysis. PCOS: polycystic ovary syndrome (*N* = 31); Control: healthy controls (*N* = 27). ^*^p ≤ 0.05; ^**^p ≤ 0.01; and ^***^p ≤ 0.001.

At the family level, the average composition and relative abundance of gut microbial communities in the two groups were shown in Figure 4A. The three dominant populations in the PCOS and the healthy controls were all Bacteroidaceae, Lachnospiraceaeand Ruminocaccaceae. As shown in Figure 4B, compared with the healthy controls, the abundance of Peptostreptococcacese and Bacteroidales S24-7 group was significantly increased in PCOS (*p* < 0.05).

At the genus level, the average composition and relative abundance of gut microbial communities in the two groups were shown in Figure 4C. The three dominant groups in PCOS and healthy controls were *Bacteroides*, *Prevotella*, and *Faecalibacterium*. As shown in Figure 4D, compared with healthy controls, the abundances of 10 genera, including *Klebsiella*, *Unclassified Peptostreptococcaceae*, and *Weissella*, were significantly higher in the PCOS, the abundance of two genera, including *Ruminococcaceae UCG 013* and *Howardella*, decreased significantly in PCOS group (all *p* < 0.05).

#### The Dominant Microflora in the Gut Microflora of PCOS and Healthy Controls

**Figure 5:**
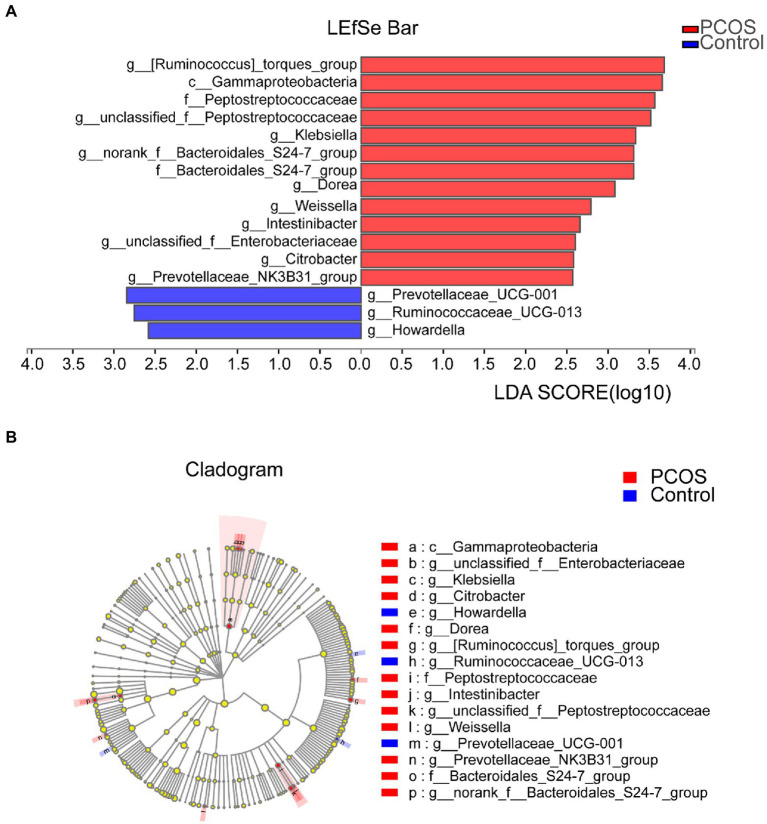
**(A)** Histogram of LDA branch of gut dominant flora in PCOS and healthy controls [LDA score (log10) > 2, *p* < 0.05]. **(B)** Phylogenetic profiles of specific bacterial groups and dominant flora between the two groups of gut microbiota. LDA, linear discriminant analysis; PCOS, polycystic ovary syndrome.

In order to determine the specific bacterial groups and dominant flora associated with PCOS, LEfSe analysis was used to show the maximum difference between the gut microflora of PCOS and healthy controls, so as to find the important gut microflora associated with PCOS. Based on LDA selection, as shown in Figures 5A, 13 bacterial groups, including *Klebsiella*, Enterobacteriaceae, and Peptostreptococcaceae, dominate in PCOS. Three bacterial groups, including *Ruminocaccaceae UCG 013* and *Prevotellaceae UCG 001*, were predominant in healthy controls. Phylogenetic profiles of specific bacterial groups and dominant flora between the two groups of gut microbiota are shown in Figure 5B.

### Differences in Gut Microflora Among PCOS Patients With Different Body Types

#### Diversity Analysis of Gut Microflora in PCOS Patients With Different Body Types

In order to analyze the structural characteristics of gut microflora in PCOS patients with different body types, 31 PCOS patients were divided into two groups according to BMI, namely the PCOSF group (BMI ≥ 24 kg/m2, *N* = 12) and PCOST group (BMI < 24 kg/m2, *N* = 19). As shown in Supplementary Figures 2A–D, according to the determination and comparison of Ace Index, Chao Index, Shannon Index, and Simpson Index, there was no significant difference in gut microbial diversity between PCOSF and PCOST, all *p* > 0.05.

We also used PCoA and NMDS to show the spatial distribution and difference degree of the gut microbial community of PCOS patients with different body types based on the distribution of gut microbial OTUs. The results are shown in Supplementary Figures 2E,F. The confidence ellipse of PCOSF and PCOST overlaps in a large area. This suggests that there is no significant difference in the distribution of gut microflora between the two groups. However, according to PCoA and NMDS, it was noted that some microbial communities still existed independently in PCOSF or PCOST. A venn diagram based on OTUs was further used to show the distribution of the two groups of gut microbial communities. The results were shown in Supplementary Figure 2G. The total abundance of OTUs in the two groups was 522, of which 389 OTUs were shared by the two groups, and 98 OTUs existed independently in the PCOST. And 35 OTUs existed independently in the PCOSF.

#### Analysis and Comparison of Gut Microflora of PCOS Patients With Different Body Types at Family and Genus Levels

To further analyze the phylogenetic status and differences of gut microflora in PCOS patients with different body types, we used the Kruskal–Wallis rank-sum test to analyze and compare the differences in the abundance of gut microorganisms in the two groups at the levels of Family and Genus.

At the family level, the average composition and relative abundance of gut microbial communities in the two groups were shown in Supplementary Figure 3A. The 4 dominant populations in the two groups were Bacteroidaceae, Lachnospiraceae, Ruminocaccaceae, and Prevotellaceae. As shown in Supplementary Figure 3B, compared with PCOST, the abundance of Peptostreptococcacese and Vellonellaceae was significantly increased in PCOSF (*p* < 0.05). There was no significant difference in the abundance of 13 families, including Ruminocaccaceae, Enterobacteriaceae, and Acidaminococcaceae, between the two groups (all *p* > 0.05).

At the genus level, the average composition and relative abundance of the two groups of gut microbial communities were shown in Supplementary Figure 3C. *Bacteroides*, *Prevotella*, *Faecalibacterium*, and *Megamonas* were the 4 dominant species in the two groups. As shown in Supplementary Figure 3D compared with PCOSF, the abundance of *Sutterella* was significantly increased in PCOST (*p* < 0.05).

#### Dominant Microflora in the Gut Microflora of PCOS With Different Body Types

In order to determine the specific bacterial groups and dominant flora associated with PCOS of different body types, LEfSe analysis was also used to show the maximum difference between PCOSF and PCOST gut microbial communities. As shown in Supplementary Figure 4A, based on LDA, 13 bacterial groups, including Veillonellaceae, Lactobacillales, and Peptostreptococcaceae, were dominant in PCOSF. Four bacterial groups, including *Candidatus Soleaferrea* and *Erysipelatoclostridium*, were dominant in PCOST. Phylogenetic profiles of specific bacterial groups and dominant flora between the two groups of gut microbiota were shown in Supplementary Figure 4B.

### Correlation Analysis Among Gut Flora, Environmental Variables, and Fecal Fatty Acids

**Figure 6:**
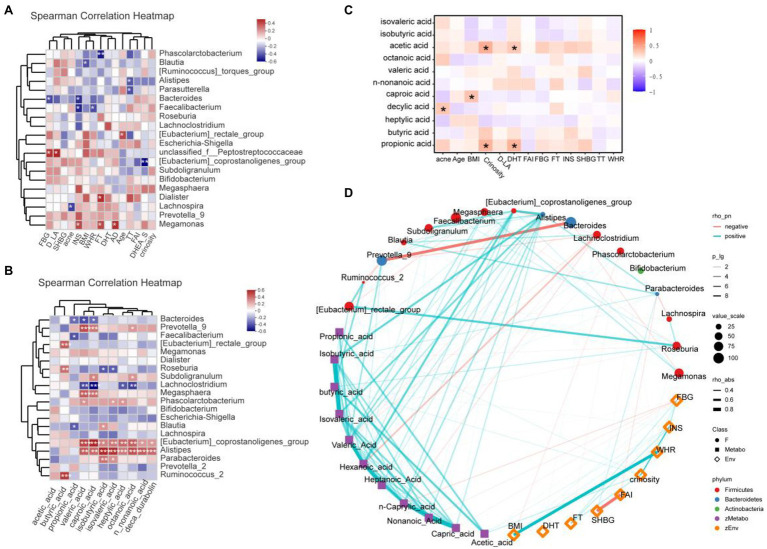
**(A)** Heatmap of correlation analysis between gut microflora and clinical indexes of PCOS. **(B)** Heatmap of correlation analysis between gut flora and SCFAs. **(C)** Heatmap of correlation analysis between fatty acids and clinical indexes. **(D)** Network diagram among clinical indicators, intestinal flora, and SCFAs. SHBG, Sex hormone binding globulin; FT, Free testosterone; DHT, Double hydrogen testosterone; FBG, Fasting blood glucose; D-LA, D-lactic acid; AD, androstenedione; DHEA-S, dehydroepiandrosterone-sulfate; INS, Insulin; BMI, Body mass index; WHR, Waist hip rate; FAI, Free androgen index; SCFAs, short-chain fatty acids. ^*^p ≤ 0.05; ^**^p ≤ 0.01; and ^***^p ≤ 0.001.

Clinical and gut permeability indexes that were significantly different between PCOS and healthy controls were selected to further analyze the related gut microbial species. The results were shown in Figure 6A. At the Genus level, *Megamonas* was positively correlated with androstenedione (AD), FT, and INS (all *p* < 0.05), while *Bacteroides* and *Faecalibacterium* were negatively correlated with INS (all *p* < 0.05), *Phascolarctobacterium* was negatively correlated with FT (*p* < 0.05).

We further analyzed the correlation between the abundance of gut flora and the fecal SCFA levels of the corresponding PCOS, and the results were shown in Figure 6B. At the genus level, *Alistipes* and *Eubateriumn coprostanoligenes group* were positively correlated with valeric acid, caproic acid, isobutyric acid, isovaleric acid, heptylic acid, octanoic acid, n-nonanoic acid, and deca durabolin (all *p* < 0.05). Though *Megasphaera and Prevotella 9* were positively correlated with valeric acid and caproic acid (all *p* < 0.05), *Lachnoclostridium* was negatively correlated with valeric acid and caproic acid (all *p* < 0.05). Butyric acid was positively correlated with *Ruminococcus 2*, *Roseburia*, and *Eubacterium rectale group* (all *p* < 0.05).

In the correlation analysis between fatty acids and laboratory indicators (Figure 6C), crinosity and DHT were positively correlated with acetic acid and propionic acid. In addition, acne was positively correlated with caproic acid, and BMI was positively correlated with decylic acid. Further, we revealed the correlation among clinical indicators, intestinal flora and SCFAs through network diagram (Figure 6D).

## Discussion

Gut flora is closely related to the occurrence and development of a variety of metabolic diseases, and each disease-related to gut flora alterations has its own relatively unique alterations in gut flora structure. For example, the prevalence of butyrate-producing bacteria was decreased in diabetic patients (Qin et al., 2012), and the abundance of Bacteroidetes was significantly decreased in obese patients, while the abundance of Actinomycetes was significantly increased (Ley et al., 2006). As a chronic endocrine and metabolic disorder, PCOS also has its unique alterations in the structure and function of gut flora, and the alterations of gut flora play an important regulatory role in the pathogenesis and evolution of PCOS (Insenser et al., 2018; Zhou et al., 2020). The results of this study showed that in PCOS, the abundance of *Ruminocaccaceae* and *Howardella* was significantly decreased, while the abundance of 13 bacterial groups, including *Klebsiella*, Peptostreptococcacese, and Gammaproteobacteria, was significantly higher than that of healthy controls, indicating that PCOS had gut flora dysregulation and unique gut flora structural changes. It is worth noting that these altered gut floras may play an important role in the pathogenesis or disease progression of PCOS. However, the significantly reduced abundance of *Ruminocaccaceae* and *Howardella* may play an important protective role in preventing the occurrence of PCOS. The abundance of Peptostreptococcaceae in PCOS increased significantly. Evidence shows that Peptostreptococcaceae may be related to the increase of cholic acid levels in patients with depression (Yu et al., 2017), and it is also involved in the gut inflammatory response. This indicates that alterations in gut flora may affect the occurrence and development of PCOS through a gut inflammatory response. Therefore, fecal bacteria transplantation to alter the composition of the intestinal microbiome of the new host is expected to be an effective treatment for PCOS. To date, there have been no clinical reports on the use of fecal bacteria transplantation for the treatment of PCOS other than in mouse models. This study provides rich basic data for further preclinical and clinical trials of fecal bacteria transplantation.

It has been speculated that gut dysbacteriosis can lead to increased gut permeability, then activate the host immune system and systemic inflammatory response, and induce insulin resistance in PCOS patients (Tremellen and Pearce, 2012). In this study, the serum levels of D-LA and DAO in PCOS patients were significantly increased compared with healthy controls, and the levels of TNF-α and LBP were also significantly increased. Studies have shown that the increase of serum D-LA level indicates the excessive proliferation of lactic acid producing bacteria in the gut, which indicates that gut dysbacteriosis exists in PCOS (Bulik-Sullivan et al., 2018). It is worth noting that DAO is an enzyme with a high concentration in the gut mucosa of humans and other mammals, which can serve as an important circulatory marker for the maturation and integrity of the gut mucosa (Luk et al., 1980), while LBP can induce a systemic inflammatory response in the body after recognizing and binding endotoxin (Rojo et al., 2007). Based on the above results, it is speculated that in PCOS, gut dysbacteriosis causes a systemic inflammatory response by affecting gut permeability changes, and then leads to the pathogenesis and progression of PCOS. In addition, inflammatory molecules can also serve as biomarkers for PCOS (Velez et al., 2021), which can aid in the diagnosis of the disease.

We further screened out the gut flora related to inflammation and gut permeability based on clinical factors TNF-α, LBP, D-LA, and DAO, and the results showed that the level of D-LA was positively correlated with *unclassified-f-Peptostreptococcaceae*. D-LA can be produced by some microorganisms or by certain metabolic pathways (Pohanka, 2020), and the abundance of *unclassified-f-Peptostreptococcaceae* is significantly altered in Graves’ disease (Jiang et al., 2021). In addition, this study found that the serum FT level was positively correlated with *Dialister* and *Megamonas*, and the TT level was negatively correlated with *Alistipes* and *Parasutterella*. Shin et al. demonstrated a significant correlation between the intestinal microbe *Megamonas* and testosterone levels in men (Shin et al., 2019). This suggests that abnormal serum androgen levels are related to gut dysbacteriosis. Therefore, abnormal androgen levels induced by PCOS may cause alterations in gut microflora homeostasis, and then alter gut function.

Dietary carbohydrates can be fermented by gut microorganisms to produce SCFAs, including acetate, propionate, butyrate, etc. (Reichardt et al., 2018). In this study, the GC–MS analysis method was used to analyze the SCFAs in stool samples of PCOS patients and healthy controls. The results showed that the contents of acetic acid and propionic acid in PCOS patients were significantly higher than those in healthy controls. Interestingly, PCOS patients also had elevated levels of acetic acid in their follicular fluid (Zhang et al., 2017). Further analysis of the correlation between gut flora and fecal fatty acids showed that the six fatty acids were positively correlated with *Eubacterium_coprostanoligenes_group* and *Alistipes*. Ahmad et al. found *Eubacterium_coprostanoligenes_group* presented increased relative abundance in obese-T2DM individuals (Ahmad et al., 2019). *Alistipes* are producers of both propionate acid and acetate, which may contribute to SCFA reduction (Polansky et al., 2015; Oliphant and Allen-Vercoe, 2019). Nogal et al. found that *Lachnoclostridium* may influence acetate circulating levels and be involved in the biosynthesis of harmful lipid compounds (Nogal et al., 2021). In this study, *Lachnoclostridiu* was negatively correlated with valeric acid, caproic acid, heptylic acid, and octanoic acid. In conclusion, the alterations of gut flora in PCOS patients may cause the metabolic alterations of SCFAs.

Obesity is one of the main clinical manifestations of PCOS. Current studies have shown that obesity is closely related to gut flora, and disorders of gut flora are common in obese people (Gomes et al., 2018). Obese children had higher rates of Firmicutes and Bacteroidetes compared with normal-weight children (Riva et al., 2017). In this study, the gut flora of obese PCOS patients and non-obese PCOS patients were compared, and the results showed that in the gut flora of PCOS patients in the obese PCOS, 13 bacterial groups, including Veillonellaceae, Lactobacillales, and Peptostreptococcaceae, were dominant. Four bacterial groups, including Candidatus Soleaferrea, *Eubacterium hallii* and Erysipelatoclostridium, were dominant in non-obese PCOS patients. These results suggest that the difference in gut flora between obese and non-obese PCOS patients is different from that between obese and healthy people, and the reason for this difference may be the impact of PCOS disease itself on gut flora. In conclusion, the gut flora of obese PCOS is different from that of non-obese PCOS, and PCOS disease itself can also affect the gut flora of patients.

In conclusion, this study comprehensively characterized the gut flora characteristics and fatty acid metabolism characteristics of PCOS patients by 16S rRNA sequencing and clinical index detection of stool samples and serum samples from participants in central China, and for the first time clarified the correlation between gut flora alterations and inflammatory indicators and gut permeability indicators, and the correlation between gut flora and short-chain fatty acid metabolism. In addition, this study is the first to comprehensively characterize the gut flora of PCOS patients with different body types. These findings will lay a solid foundation for the pathogenesis of PCOS and provide some help for intervention including fecal bacteria transplantation.

## Data Availability Statement

The data presented in the study are deposited in the European Bioinformatics Institute European Nucleotide Archive repository, accession number PRJNA823867.

## Ethics Statement

The studies involving human participants were reviewed and approved by The First Affiliated Hospital of Zhengzhou University (no. 2021-KY-0069). The patients/participants provided their written informed consent to participate in this study.

## Author Contributions

GL and GQ designed the study. ZL, FR, and HS collated and analyzed the clinical data of the subjects. QZ, YS, XF, and XM were involved in verifying whether subjects were eligible to participate in the study. GL and ZL analyzed the data. GL wrote the manuscript. GQ revised the manuscript. All authors contributed to the article and approved the submitted version.

## Conflict of Interest

The authors declare that the research was conducted in the absence of any commercial or financial relationships that could be construed as a potential conflict of interest.

## Publisher’s Note

All claims expressed in this article are solely those of the authors and do not necessarily represent those of their affiliated organizations, or those of the publisher, the editors and the reviewers. Any product that may be evaluated in this article, or claim that may be made by its manufacturer, is not guaranteed or endorsed by the publisher.

## Footnotes

^1^ http://www.R-project.org/

^2^ http://huttenhower.sph.harvard.edu/lefse/

## References

[ref1] Ahmad A. Yang W. Chen G. Shafiq M. Javed S. Ali Zaidi S. S.. (2019). Analysis of gut microbiota of obese individuals with type 2 diabetes and healthy individuals. PLoS One 14:e0226372. doi: 10.1371/journal.pone.0226372, PMID: 31891582 PMC6938335

[ref2] Azziz R. Carmina E. Chen Z. Dunaif A. Laven J. S. Legro R. S.. (2016). Polycystic ovary syndrome. Nat. Rev. Dis. Primers. 2:16057. doi: 10.1038/nrdp.2016.57 27510637

[ref3] Baker J. M. Al-Nakkash L. Maturitas M. M. H.-K. J. (2017). Estrogen-gut microbiome axis. Physiological and clinical implications 103, 45–53. doi: 10.1016/j.maturitas.2017.06.025, PMID: 28778332

[ref4] Brown C. T. Davis-Richardson A. G. Giongo A. Gano K. A. Crabb D. B. Mukherjee N.. (2011). Gut microbiome metagenomics analysis suggests a functional model for the development of autoimmunity for type 1 diabetes. PLoS One 6:e25792. doi: 10.1371/journal.pone.0025792, PMID: 22043294 PMC3197175

[ref5] Bulik-Sullivan E. C. Roy S. Elliott R. J. Kassam Z. Lichtman S. N. Carroll I. M.. (2018). Intestinal Microbial and Metabolic Alterations Following Successful Fecal Microbiota Transplant for D-Lactic Acidosis. J. pediatric gastroenterology and nutrition 67, 483–487. doi: 10.1097/mpg.0000000000002043 29901551

[ref6] Canfora E. E. Meex R. C. R. Venema K. Blaak E. E. (2019). Gut microbial metabolites in obesity, NAFLD and T2DM. Nat. Rev. Endocrinol. 15, 261–273. doi: 10.1038/s41574-019-0156-z, PMID: 30670819

[ref7] Duan L. An X. Zhang Y. Jin S. Zhao R. Zhou Y.. (2021). Gut microbiota as the critical correlation of polycystic ovary syndrome and type 2 diabetes mellitus. Biomed. Pharmacother. 142:112094. doi: 10.1016/j.biopha.2021.112094, PMID: 34449321

[ref8] Escobar-Morreale H. F. (2018). Polycystic ovary syndrome: definition, aetiology, diagnosis and treatment. Nat. Rev. Endocrinol. 14, 270–284. doi: 10.1038/nrendo.2018.24, PMID: 29569621

[ref9] Gill S. R. Pop M. Deboy R. T. Eckburg P. B. Turnbaugh P. J. Samuel B. S.. (2006). Metagenomic analysis of the human distal gut microbiome. Science 312, 1355–1359. doi: 10.1126/science.1124234, PMID: 16741115 PMC3027896

[ref10] Gomes A. C. Hoffmann C. Mota J. F. (2018). The human gut microbiota: metabolism and perspective in obesity. Gut Microbes 9, 1–18. doi: 10.1080/19490976.2018.1465157, PMID: 29667480 PMC6219651

[ref11] Insenser M. Murri M. Campo R. D. Martínez-García M. Á. Fernández-Durán E. Escobar-Morreale H. F. (2018). Gut microbiota and the polycystic ovary syndrome: influence of sex, sex hormones, and obesity. The J. Clinical Endocrinology & Metabol. 103, 2552–2562. doi: 10.1210/jc.2017-02799, PMID: 29897462

[ref12] Jayasena C. N. Franks S. (2014). The management of patients with polycystic ovary syndrome. Nat. Rev. Endocrinol. 10, 624–636. doi: 10.1038/nrendo.2014.102 25022814

[ref13] Jiang W. Yu X. Kosik R. O. Song Y. Qiao T. Tong J.. (2021). Gut microbiota may play a significant role in the pathogenesis of Graves' disease. Thyroid 31, 810–820. doi: 10.1089/thy.2020.0193, PMID: 33234057 PMC8110022

[ref14] Kumarendran B. O'Reilly M. W. Manolopoulos K. N. Toulis K. A. Gokhale K. M. Sitch A. J.. (2018). Polycystic ovary syndrome, androgen excess, and the risk of nonalcoholic fatty liver disease in women: A longitudinal study based on a United Kingdom primary care database. PLoS Med. 15:e1002542. doi: 10.1371/journal.pmed.1002542, PMID: 29590099 PMC5873722

[ref15] Kusamoto A. Harada M. Azhary J. M. K. Kunitomi C. Nose E. Koike H.. (2021). Temporal relationship between alterations in the gut microbiome and the development of polycystic ovary syndrome-like phenotypes in prenatally androgenized female mice. FASEB J. 35:e21971. doi: 10.1096/fj.202101051R, PMID: 34653284

[ref16] Ley R. Peterson D. Gordon J. J. C. (2006). Ecological and evolutionary forces shaping microbial diversity in the human intestine. Cell 124, 837–848. doi: 10.1016/j.cell.2006.02.017 16497592

[ref17] Liang Z. Di N. Li L. Yang D. (2021). Gut microbiota alterations reveal potential gut-brain axis changes in polycystic ovary syndrome. J. Endocrinol. Investig. 44, 1727–1737. doi: 10.1007/s40618-020-01481-5, PMID: 33387350

[ref18] Lin W. Wen L. Wen J. Xiang G. (2021). Effects of sleeve Gastrectomy on fecal gut microbiota and short-chain fatty acid content in a rat model of polycystic ovary syndrome. Front Endocrinol (Lausanne) 12:747888. doi: 10.3389/fendo.2021.747888, PMID: 34858330 PMC8631770

[ref19] Lindheim L. Bashir M. Münzker J. Trummer C. Zachhuber V. Leber B.. (2017). Alterations in gut microbiome composition and barrier function are associated with reproductive and metabolic defects in women with polycystic ovary syndrome (PCOS): A pilot study. PLoS One 12:e0168390. doi: 10.1371/journal.pone.0168390, PMID: 28045919 PMC5207627

[ref20] Liu R. Zhang C. Shi Y. Zhang F. Li L. Wang X.. (2017). Dysbiosis of gut microbiota associated with clinical parameters in polycystic ovary syndrome. Front. Microbiol. 8:324. doi: 10.3389/fmicb.2017.00324, PMID: 28293234 PMC5328957

[ref21] Luk G. D. Bayless T. M. Baylin S. B. (1980). Diamine oxidase (histaminase). A circulating marker for rat intestinal mucosal maturation and integrity. J. Clin. Invest. 66, 66–70. doi: 10.1172/jci109836, PMID: 6772669 PMC371506

[ref22] Macfarlane G. Macfarlane S. (1997). Human colonic microbiota: ecology, physiology and metabolic potential of intestinal bacteria. Scandinavian J. Gastroenterology 222, 3–9. doi: 10.1080/00365521.1997.11720708 9145437

[ref23] Mitreva M. Human Microbiome Project Consortium (2012). Structure, Function and Diversity of the Healthy human Microbiome. Nature 486, 207–214. doi: 10.1038/nature11234, PMID: 22699609 PMC3564958

[ref24] Nenci A. Becker C. Wullaert A. Gareus R. van Loo G. Danese S.. (2007). Epithelial NEMO links innate immunity to chronic intestinal inflammation. Nature 446, 557–561. doi: 10.1038/nature05698 17361131

[ref25] Nogal A. Louca P. Zhang X. Wells P. M. Steves C. J. Spector T. D.. (2021). Circulating levels of the short-chain fatty acid acetate mediate the effect of the gut microbiome on visceral fat. Front. Microbiol. 12:711359. doi: 10.3389/fmicb.2021.711359, PMID: 34335546 PMC8320334

[ref26] Oksanen J.. (2011). vegan: Community Ecology Package. R package version 1.17–9. Available at: http://cran.r-project.org/package= vegan (Accessed October 25, 2018).

[ref27] Oliphant K. Allen-Vercoe E. (2019). Macronutrient metabolism by the human gut microbiome: major fermentation by-products and their impact on host health. Microbiome 7:91. doi: 10.1186/s40168-019-0704-8, PMID: 31196177 PMC6567490

[ref28] Palomba S. Piltonen T. T. Giudice L. C. (2021). Endometrial function in women with polycystic ovary syndrome: a comprehensive review. Hum. Reprod. Update 27, 584–618. doi: 10.1093/humupd/dmaa051, PMID: 33302299

[ref29] Pickard J. Chervonsky A. V. (2015). Intestinal fucose as a mediator of host-microbe symbiosis. The J. Immun. 194, 5588–5593. doi: 10.4049/jimmunol.1500395, PMID: 26048966 PMC4536407

[ref30] Pohanka M. (2020). D-lactic acid as a metabolite: toxicology, diagnosis, and detection. Biomed. Res. Int. 2020, 3419034–3419039. doi: 10.1155/2020/3419034, PMID: 32685468 PMC7320276

[ref31] Polansky O. Sekelova Z. Faldynova M. Sebkova A. Sisak F. Rychlik I. (2015). Important metabolic pathways and biological processes expressed by chicken Cecal microbiota. Appl. Environ. Microbiol. 82, 1569–1576. doi: 10.1128/aem.03473-15, PMID: 26712550 PMC4771310

[ref32] Qin J. Li Y. Cai Z. Li S. Zhu J. Zhang F.. (2012). A metagenome-wide association study of gut microbiota in type 2 diabetes. Nature 490, 55–60. doi: 10.1038/nature11450 23023125

[ref33] Reichardt N. Vollmer M. Holtrop G. Farquharson F. M. Wefers D. Bunzel M.. (2018). Specific substrate-driven changes in human faecal microbiota composition contrast with functional redundancy in short-chain fatty acid production. The ISME Journal 12, 610–622. doi: 10.1038/ismej.2017.196, PMID: 29192904 PMC5776475

[ref34] Riva A. Borgo F. Lassandro C. Verduci E. Morace G. Borghi E.. (2017). Pediatric obesity is associated with an altered gut microbiota and discordant shifts in Firmicutes populations. Environ. Microbiol. 19, 95–105. doi: 10.1111/1462-2920.13463, PMID: 27450202 PMC5516186

[ref35] Rojo O. P. Román A. L. S. Arbizu E. A. Martínez A. H. Sevillano E. R. Martínez A. A.. (2007). Serum lipopolysaccharide-binding protein in endotoxemic patients with inflammatory bowel disease, Inflammatory bowel diseases, 13, 269–277. doi: 10.1002/ibd.20019 17206721

[ref36] Rotterdam E. A.-S. P. C. W. G. (2004). Revised 2003 consensus on diagnostic criteria and long-term health risks related to polycystic ovary syndrome. Fertil. Steril. 81, 19–25. doi: 10.1016/j.fertnstert.2003.10.004, PMID: 14711538

[ref37] Shin J. H. Park Y. H. Sim M. Kim S. A. Joung H. Shin D. M. (2019). Serum level of sex steroid hormone is associated with diversity and profiles of human gut microbiome. Res. Microbiol. 170, 192–201. doi: 10.1016/j.resmic.2019.03.003, PMID: 30940469

[ref38] Smith C. A. Want E. J. O'Maille G. Abagyan R. Siuzdak G. (2006). XCMS: processing mass spectrometry data for metabolite profiling using nonlinear peak alignment, matching, and identification. Anal. Chem. 78, 779–787. doi: 10.1021/ac051437y, PMID: 16448051

[ref39] Teede H. Deeks A. Moran L. (2010). Polycystic ovary syndrome: a complex condition with psychological, reproductive and metabolic manifestations that impacts on health across the lifespan. BMC Med. 8:41. doi: 10.1186/1741-7015-8-41, PMID: 20591140 PMC2909929

[ref40] Teede H. Misso M. Tassone E. C. Dewailly D. Ng E. H. Azziz R.. (2019). Anti-Mullerian hormone in PCOS: A review informing international guidelines. Trends Endocrinol. Metab. 30, 467–478. doi: 10.1016/j.tem.2019.04.006, PMID: 31160167

[ref41] Tremellen K. Pearce K. (2012). Dysbiosis of gut microbiota (DOGMA) – A novel theory for the development of polycystic ovarian syndrome. Med. Hypotheses 79, 104–112. doi: 10.1016/j.mehy.2012.04.016, PMID: 22543078

[ref42] Velez L. M. Seldin M. Motta A. B. (2021). Inflammation and reproductive function in women with polycystic ovary syndrome†. Biol. Reprod. 104, 1205–1217. doi: 10.1093/biolre/ioab050, PMID: 33739372 PMC8785941

[ref43] Yang Y. L. Zhou W. W. Wu S. Tang W. L. Wang Z. W. Zhou Z. Y.. (2021). Intestinal Flora is a key factor in insulin resistance and contributes to the development of polycystic ovary syndrome. Endocrinology 162:b118. doi: 10.1210/endocr/bqab118, PMID: 34145455 PMC8375444

[ref44] Yu M. Jia H. Zhou C. Yang Y. Zhao Y. Yang M.. (2017). Variations in gut microbiota and fecal metabolic phenotype associated with depression by 16S rRNA gene sequencing and LC/MS-based metabolomics. J. pharmaceutical and biomedical analysis 138, 231–239. doi: 10.1016/j.jpba.2017.02.008 28219800

[ref45] Zeng X. Huang Y. Zhang M. Chen Y. Ye J. Han Y.. (2022). Anti-Müllerian hormone was independently associated with central obesity but not with general obesity in women with PCOS. Endocr. Connect 11:0243. doi: 10.1530/ec-21-0243, PMID: 34822342 PMC8789017

[ref46] Zeng X. Huang Y. Zhang M. Yun C. Jiawen Y. Han Y.. (2021). AMH was independently associated with central obesity but not with general obesity in women with PCOS. Endocr. Connect. 11:0243. doi: 10.1530/EC-21-0243, PMID: 34822342 PMC8789017

[ref47] Zhang Y. Liu L. Yin T. L. Yang J. Xiong C. L. (2017). Follicular metabolic changes and effects on oocyte quality in polycystic ovary syndrome patients. Oncotarget 8, 80472–80480. doi: 10.18632/oncotarget.19058, PMID: 29113318 PMC5655213

[ref48] Zhou L. Ni Z. Cheng W. Yu J. Sun S. Zhai D.. (2020). Characteristic gut microbiota and predicted metabolic functions in women with PCOS. Endocrine Connections 9, 63–73. doi: 10.1530/ec-19-0522, PMID: 31972546 PMC6993273

