# Supplementary material for: Alterations of Gut Microbiome and Fecal Fatty Acids in Patients With Polycystic Ovary Syndrome in Central China
Source: Front Microbiol. 2022 Jul 1;13:911992. doi: 10.3389/fmicb.2022.911992 (PMC9283120; doi:10.3389/fmicb.2022.911992)
Supplement: Supplementary file 1 [file Data_Sheet_1.docx]

**Alterations in Gut Microbiota and Fatty Acid Metabolism in Patients with Polycystic Ovary Syndrome**

**Supplement methods**

**Research object**

From 2021 to 2022, 31 patients with PCOS who visited the department of Gynecology of the First Affiliated Hospital of Zhengzhou University and 27 healthy volunteers who underwent physical examination in the Department of Physical Examination of the First Affiliated Hospital of Zhengzhou University were recruited. This study was discussed and approved by the Ethics Committee of the First Affiliated Hospital of Zhengzhou University (Approval No. 2021-KY-0069). All the selected participants understood the risks and benefits of this study and signed informed consent.

**Participant inclusion criteria**

a) Aged between 18 and 35; b) PCOS patients should meet the Rotterdam diagnostic criteria: sparse ovulation or anovulation; Clinical manifestations of hyperandrogen and/or hyperandrogenemia; Polycystic ovary: ≥ 12 follicles with a diameter of 2-9 mm on one or both ovaries, and/or ovarian volume ≥ 10 mL. Two of the above three criteria were met, and other diseases causing Kaohsiung and ovulation disorders were excluded. c) The healthy volunteers had regular menstrual cycles, ranging from 28 to 35 days.

**Participant exclusion criteria**

a) Have used antibacterial drugs, antifungal drugs or non-steroidal anti-inflammatory drugs in the last 3 months; b) steroid hormones, hypoglycemic drugs and antihypertensive drugs were used in the last 3 months; c) Have taken prebiotics, probiotics, synbiotics and other microecological regulation products in the past 2 weeks; d) History of major gastrointestinal surgery in recent 5 years; e) Chronic diseases of the gastrointestinal tract, such as Inflammatory bowel disease (IBD), including ulcerative colitis, Crohn's disease, or uncertain colitis; f) persistent and infectious gastroenteritis, colitis or gastritis; Persistent or chronic diarrhea of unknown etiology; Clostridium difficile infection (recurrent) or Helicobacter pylori infection (untreated); g) Historical gastric or intestinal ulcers/gastrointestinal bleeding, gastrointestinal polyps, masses, dysplasia or cancer; h) Irritable bowel syndrome; i) Suffering from diseases or factors related to gut microbes, such as diabetes, cardiovascular disease, autism, alcoholism, immune deficiency, etc.; j) pregnant women and lactating women; k) Carrying hepatitis B/C virus antigen.

**Extraction and purification of gut microbe DNA**

a) Suck 200 μL stool liquid sample into 2 mL centrifuge tube and place it on ice; b) Add buffer SA 500 μL, buffer SC 100 μL, protease K 15 μL into 2 mL centrifuge tube, add grinding beads 0.25 g, repeatedly shock the sample for 1 min, then use TGrinder H24 tissue grinding homogenizer to fully mix the sample; c) It was cracked at 95℃ water bath for 15 min, during which it was shaken and mixed for 3 times; d) The samples were mixed, centrifuged at 12,000 rpm for 3 min, and the supernatant was collected. 100 μL RNase A was added to the supernatant, and the mixture was mixed and let stand at room temperature for 5 min; e) Add buffer SH 200 μL to supernatant, mix well, and stand on ice for 5 min; f) Centrifuge at 12,000 rpm for 3 min, collect supernatant, transfer 500 μL supernatant to a new centrifuge tube; Add 500 μL buffer GFA into the centrifuge tube; g) DNA extract was purified by CR2 adsorption column, the extract was added to the adsorption column, centrifuged at 12,000 rpm for 30 s, and the adsorption column was retained; h) Put the adsorption column into a new centrifugal tube, add buffer GD 500 μL, centrifuge (12,000 rpm) for 30 s, retain the adsorption column; i) The adsorption column was placed in a new centrifugal tube, PW 700 μL was added, centrifuged at 12,000 rpm for 30 s, and the adsorption column was retained, repeated once; j) Place the adsorption column in a new centrifugal tube, centrifuge again, and dry the adsorption column at room temperature; k) DNA was collected, and the adsorption column was placed in a new centrifugal tube, and eluting buffer (TB 50 μL) was dropped into the center. After standing at room temperature for 5 min, the DNA was collected and purified by centrifugation (12,000 rpm) for 2 min.

**PCR amplification**

Specific primers with barcode were used to amplify the v3-v4 variable region of 16S rRNA gene. The primers used were shown in **Table 1**, the PCR reaction system was shown in **Table 2**, and the PCR reaction procedure was shown in **Table 3**.

| **PCR** | **Primer sequence（5’-3’）** |
| --- | --- |
| ***Outer PCR*** | |
| Forwrad | AATGATACGGCGACCACCGAGATCTACAC(Index1)  ACACTCTTTCCCTACACGACG |
| Reverse | CAAGCAGAAGACGGCATACGAGAT(Index2)  GTGACTGGAGTTCAGACGTGTGCTCTTCCGATCT |
| ***Inner PCR*** | |
| Forwrad | ACACTCTTTCCCTACACGACGCTCTTCCGATCTCCTACGGGNGGCWGCAG |
| Reverse | AGACGTGTGCTCTTCCGATCTGACTACHVGGGTATCTAATCC |

**Table 1. 16S rRNA V3-V4 PCR specific primer sequences.**

| Reagent | Volume |
| --- | --- |
| Forward primer（5 μM） | 0.5 μL |
| Reverse primer（5 μM） | 0.5 μL |
| 5 × FastPfu Buffer | 4 μL |
| FastPfu Polymerase | 0.4 μL |
| 2.5 mM dNTPs | 2 μL |
| BSA | 0.2 μL |
| Template DNA | 10 ng |
| ddH_2_O | Complement reaction system to 20 μL |

**Table 2. 16S rRNA V3-V4 PCR reaction system.**

| Temperature | Time | Recurring number |
| --- | --- | --- |
| 95^o^C | 3 min | 1 |
| 95^o^C | 30 s | 27 |
| 55^o^C | 30 s |  |
| 72^o^C | 45 s |  |
| 72^o^C | 10 min | 1 |

**Table 3. 16S rRNA V3-V4 PCR reaction procedure.**

**Miseq sequencing**

The splice sequence of DNA fragment is complementary to the base sequence embedded on the chip and fixed on the chip. The DNA fragment is used as template and the base sequence fixed on the chip is used as primer for PCR synthesis, and the target DNA fragment to be tested is synthesized on the chip. After denaturation and annealing, the other end of the DNA fragment on the chip was randomly complementary with another primer nearby, which was also fixed, forming a bridge, PCR amplification, DNA clusters, DNA amplifiers linearized into single chains, adding the modified DNA polymerase and dNTP with four fluorescence markers, and only one base was synthesized each cycle. By scanning the reaction plate with laser, it was shown that the nucleotide species polymerized in the first reaction of each template sequence were read. The fluorescein group and the terminator group were chemically cleaved to restore the viscosity of the 3 'end and continue to polymerize the second nucleotide. The results of fluorescence signal collected in each round were counted and the sequence of template DNA fragment was obtained.

**Data Processing**

First, FASTP software (<https://github.com/OpenGene/fastp>, version 0.20.0) was used for quality control of the original sequencing sequence. Then, FLASH (<http://www.cbcb.umd.edu/software/flash>, version 1.2.7) software is used to splice the sequence as follows: (1) Filter reads with tail mass values below 20 bases and set a 50bp window. If the average quality value in the window is lower than 20, the back-end base is cut off from the window. The reads below 50bp after quality control are filtered, and the reads containing N base are removed. (2) According to the overlap relation between PE reads, the pairs of reads are spliced into a sequence. The minimum length overlap is 10bp. (3) The maximum error matching ratio allowed by the overlap area of the stitching sequence is 0.2. Remove nonconforming sequences. (4) Samples were differentiated according to barcode and primers at the beginning and end of the sequence, and the sequence direction was adjusted. The allowed mismatch number of barcode is 0, and the maximum primer mismatch number is 2.

UPARSE software (<http://drive5.com/uparse/>, version 7.1) was used to perform OTU clustering for sequences according to 97% similarity, and the specific process was as follows: (1) Nonrepeating sequences were extracted from optimized sequences and single sequences without repetition were removed. (2) OTU clustering was performed for non-repeating sequences (excluding single sequences) according to 97% similarity. Chimeras were removed in the clustering process and OTU representative sequences were obtained. (3) Map all optimized sequences to OTU representative sequences, and select sequences with more than 97% similarity to OTU representative sequences to generate OTU tables.

Finally, the RDP classifier (<http://rdp.cme.msu.edu/>, version 2.2) was used to annotate each sequence for species classification. The comparison database was the Silva 16S rRNA database (V138), and the comparison threshold was set to 70%.

**Pretreatment of human faecal fatty acid test samples**

Take 200 μL sample into 2 mL EP tube, add 0.5 mL H_2_O, vortex shock for 10 s, make the sample fully mixed. Steel balls were added to the sample, and the tissue grinder was used to grind the sample at a frequency of 35HZ for 4 min to homogenize the sample, followed by ultrasound in the ice water domain for 5 min. The samples were centrifuged at 4℃ and 5,000 rpm for 20 min, 0.4 mL supernatant was collected into a new 2 mL EP tube, 0.5 mL H_2_O was added to dilute the sample, and the vortex shook for 10 s to make the sample fully mixed. The samples were ground for 4 min at 35HZ frequency, and ultrasound was performed for 5 min in ice water domain. The samples were then centrifuged at 4℃ and 5,000 rpm for 20 min. 0.8mL supernatant was merged into 2 mL EP tube, and 0.1 mL 50% H_2_SO_4_ and 0.5 mL internal standard solution (2-methylpentanoic acid: 25 mg/L; Methyl tert-butyl ether: 25 mg/L), vortex oscillation 10 s; The samples were centrifuged at 4℃ and 12,000 rpm for 15 min and stood at 20℃ for 30 min. The supernatant was collected into the injection bottle for GC-MS analysis.

**GC-MS detection**

The production of acetic acid, propionic acid, isobutyric acid, butyric acid, isovaleric acid, valeric acid, hexanoic acid, heptanic acid, octanic acid, nonanoic acid and decanoic acid in human feces were analyzed by Agilent 7890B gas chromatography and Agilent 5977B mass spectrometry.

Agilent HP-FFAP capillary column (30 m × 250 μm × 0.25 μm) was used. The carrier gas was helium, and the injection volume was set to 1 μL. The solvent delay time was set to 5 min. The column flow rate was 1 mL/min. The heating procedure of the column box was as follows: 80℃ for 1 min, heating to 150℃ at a rate of 5℃/min, heating to 230℃ at a rate of 40℃/min, and maintaining at 230℃ for 12 min; The injector temperature and the transmission line temperature were 240℃, the ion source temperature was 230℃, the four-stage rod temperature was 150℃, the ionization voltage was -70 eV, and the scanning range of mass spectrum was M/Z 33-200.

Data processing

In this study, 11 peaks were detected and 11 metabolites were left after relative standard deviation de-noising. Then, the missing values were filled up by the median value. The final dataset containing the information of peak number, sample name, and normalized peak area was imported to SIMCA15.0.2 software package (Sartorius Stedim Data Analytics AB, Umea, Sweden) for multivariate analysis. Data were scaled and logarithmic transformed to minimize the impact of both noise and high variance of the variables. Furthermore, Spearman correlation analysis was used to conduct correlation analysis on fatty acids, intestinal flora, and laboratory indicators of the same individual.

**Detection of IL-6 and TNF-α**

Serum IL-6 and TNF-α were detected using Human IL-6/TNF-α ELISA Kit.

A) The samples and kits were balanced at room temperature for 30 min, and mixed evenly using IKA MS3 vortex oscillator. The following reagents were configured on ice: a). Cytokine standard: Lyophilized powder is dissolved and left to rest for 10 min. Dilution of the standard at a concentration of 25 pg/mL, 50 pg/mL, 100 pg/mL, 200 pg/mL, 400 pg/mL and 800 pg/mL is done using an Dilution buffer R (1 ×); b). Biotin-labeled antibody: Dilute the biotin-labeled antibody against IL-6 and TNF-α in a 1:50 Dilution Dilution Dilution buffer to form a 1 × primary antibody working solution; c). Horse Radish Peroxidase (HRP) labeled streptaavidin: Dilute 1 × secondary antibody working solution in a Dilution buffer of 1:100; d). Washing buffer (1 ×): Dilute the Washing buffer (50 ×) with distilled water in a ratio of 1:50.

B) Perform immunodetection on serum samples as follows: a). After the blood samples were set for 20 min, centrifuged for 20 min, supernatant was collected and stored at -4℃ as samples to be tested, or stored at -20℃; b). Add 100 μL of standard solution and sample to each reaction well in a 96-well plate, and add 100 μL Dilution buffer R (1 ×) to the control well；c). Add 50 μL biotin-labeled antibody (1 ×) to each well, mix well, seal with plate membrane, and react at room temperature for 3 h; d). Remove the liquid in the reaction well, add 300 μL Washing buffer (1 x) to each well, stand for 1 min, remove the Washing buffer, repeat 3 times, and drain the excess liquid with filter paper; e). Add 100 μL HRP-labeled streptavidin to each well, seal with sealing plate membrane, and react at room temperature for 20 min；f). Repeat operation d; g). Add 100 μL TMB to each well, react at room temperature and away from light for 30 min; h). After the reaction, add 100 μL reaction stop solution; i). 450nm absorbance was measured using Thermo Scientific™ Microplate reader; j). Il-6 and TNF-α concentrations were calculated according to the standard curve.

**Detection of LBP and DAO**

Serum LBP and DAO levels were determined using human LBP and DAO elisa kits.

A) The samples and kits were balanced for 30 min at room temperature, and mixed evenly with IKA MS3 vortex oscillator. The following reagents were configured on ice: a). Standard dilution: mix 120 μL standard dilution with 120 μL original standard to get 40 ng/mL standard solution. Dilute the standard at the following concentrations: 2.5 ng/mL, 5 ng/mL, 10 ng/mL, 20 ng/mL and 50 ng/mL; b). Wash solution: dilute and concentrate the wash solution 30 times with distilled water.

B) Immunodetection of LBP and DAO in serum samples was performed as follows: a). After the blood samples were set for 20 min, centrifuged for 20 min, supernatant was collected and stored at -4℃ as samples to be tested, or stored at -20℃; b). Add 50 μL standard working solution or 40 μL sample to each reaction well in the 96-well plate, and do not add any liquid to the blank control well; c). Add 50 μL HRP labeled streptavidin to the standard well, the standard solution already contains biotin antibody, add 10 μL LBP antibody or DAO antibody, 50 μL HRP labeled streptavidin to the sample well, seal the 96-well plate with sealing plate membrane, mix well, reaction at 37℃ for 60 min, No antibody was added to the blank hole; d). Remove sealing plate membrane, remove liquid, add 500 μL washing solution (30 ×), rest for 1 min, remove liquid, repeat 5 times, and drain the excess liquid with filter paper; e). Add 50 μL chromogenic agent A and chromogenic agent B to each well, shake and mix, at 37℃, and develop color away from light for 15 min; f). 450nm absorbance was measured using Thermo Scientific™ Microplate reader; g). Calculate LBP and DAO concentrations according to standard curves.

**Detection of D-lactic acid（D-LA）**

The levels of D-LA in serum of PCOS patients and healthy females in the control group were measured using a colorimetric assay kit.

A) The samples and kits were balanced for 30 min at room temperature, and mixed evenly with IKA MS3 vortex oscillator. The following reagents were configured on ice: a). D-LA enzyme mixture: Dissolve d-LA freeze-dried powder into 0.22 mL D-LA detection buffer to make it evenly mixed; b). Matrix mixture: Dissolve the lyophilized powder of D-LA reaction matrix into 0.22 mL D-LA detection buffer to make it evenly mixed; c). Add 10 μL 100 mM D-LA standard solution to 990 μL detection buffer, mix well, add 0, 2, 4, 6, 8, 10 μL standard solution to each well, add 50 μL, 48 μL, 46 μL, 44 μL, 42 μL, 50 μL detection buffer to each well accordingly. The concentrations of D-La in each well were 0 nmol/mL, 40 nmol/mL, 80 nmol/mL, 120 nmol/mL, 160 nmol/mL and 200 nmol/mL, respectively; d). Add 1 μL, 5 μL, 10 μL, 25 μL and 50 μL to each well, and fill the volume with detection buffer solution to 50 μL; e). Prepare reaction mixture, mix 46 μL D-LA detection buffer, 2 μL matrix mixture, and 2 μL D-La enzyme mixture.

B) Colorimetric detection of D-LA in serum samples was performed as follows: a). Add 50 μL reaction mixture to different concentrations of standard and sample, mix well; b). Reaction at room temperature for 30 min; c). Determine 450 nm absorbance using Thermo Scientific™ Microplate reader; d). Calculate D-LA concentration according to the standard curve.
