# Supplementary material for: Alterations of Gut Microbiome and Fecal Fatty Acids in Patients With Polycystic Ovary Syndrome in Central China
Source: Front Microbiol. 2022 Jul 1;13:911992. doi: 10.3389/fmicb.2022.911992 (PMC9283120; doi:10.3389/fmicb.2022.911992)
Supplement: Supplementary file 2 [file Data_Sheet_2.docx]

**Alterations in Gut Microbiota and Fatty Acid Metabolism in Patients with Polycystic Ovary Syndrome** **in Central China**

**Supplementary Tables**

**Table 1. Standard curves and retention time of substances**

| **Number** | **Component** | **Retention time（min）** | | **Linear equation** | **Correlation coefficient R^2^** | **Linearity range（mg/L）** |
| --- | --- | --- | --- | --- | --- | --- |
| 1 | Acetic acid | 6.981 | y=0.0112x+0.001 | | 1.000 | 0.5~100 |
| 2 | Propionic acid | 8.630 | y=0.011x+0.0009 | | 0.9999 | 0.5~100 |
| 3 | Isobutyric acid | 9.184 | y=0.0209x-0.0006 | | 1.000 | 0.5~20 |
| 4 | Butyric acid | 10.459 | y=0.0267x+0.0292 | | 0.9995 | 0.5~200 |
| 5 | Isovaleric acid | 11.297 | y=0.0326x+0.0001 | | 0.9998 | 0.5~20 |
| 6 | Valeric acid | 12.744 | y=0.0341x-0.0048 | | 0.9999 | 0.5~20 |
| 7 | Hexanoic acid | 14.950 | y=0.0237x-0.0015 | | 0.9999 | 0.02~20 |
| 8 | Heptanoic acid | 16.255 | y=0.0204x-0.0016 | | 0.9998 | 0.02~20 |
| 9 | Octanoic acid | 16.963 | y=0.0175x-0.0017 | | 0.9998 | 0.02~20 |
| 10 | Pelargonic acid | 17.514 | y=0.0142x-0.0006 | | 0.9999 | 0.02~20 |
| 11 | Decylic acid | 18.038 | y=0.0129x-0.0009 | | 0.9999 | 0.02~20 |

**Table 2. GC-MS QC information table**

| **Number** | **Component** | **QC1（mg/L）** | **Recovery rate（%）** | **QC7（mg/L）** | **Recovery rate（%）** |
| --- | --- | --- | --- | --- | --- |
| 1 | Acetic acid | 9.84 | 98.4 | 9.96 | 99.6 |
| 2 | Propionic acid | 10.29 | 102.9 | 10.40 | 104.0 |
| 3 | Isobutyric acid | 10.18 | 101.8 | 10.10 | 101.0 |
| 4 | Butyric acid | 9.92 | 99.2 | 10.01 | 100.1 |
| 5 | Isovaleric acid | 10.02 | 100.2 | 100.6 | 100.6 |
| 6 | Valeric acid | 10.20 | 102.0 | 10.04 | 100.4 |
| 7 | Hexanoic acid | 10.11 | 101.1 | 10.16 | 101.6 |
| 8 | Heptanoic acid | 10.10 | 101.0 | 10.68 | 106.8 |
| 9 | Octanoic acid | 10.11 | 101.1 | 10.50 | 105.0 |
| 10 | Pelargonic acid | 10.02 | 100.2 | 10.71 | 107.1 |
| 11 | Decylic acid | 9.97 | 99.7 | 10.56 | 105.6 |

**Supplementary Figure**


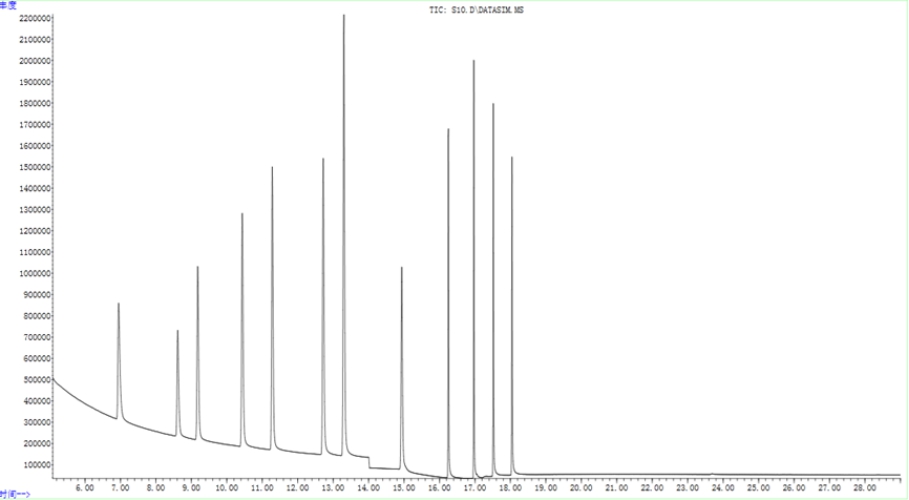


**Supplementary Figure 1.** Ion flow diagram of target compound standard GC-MS SIM.

**Supplementary** **
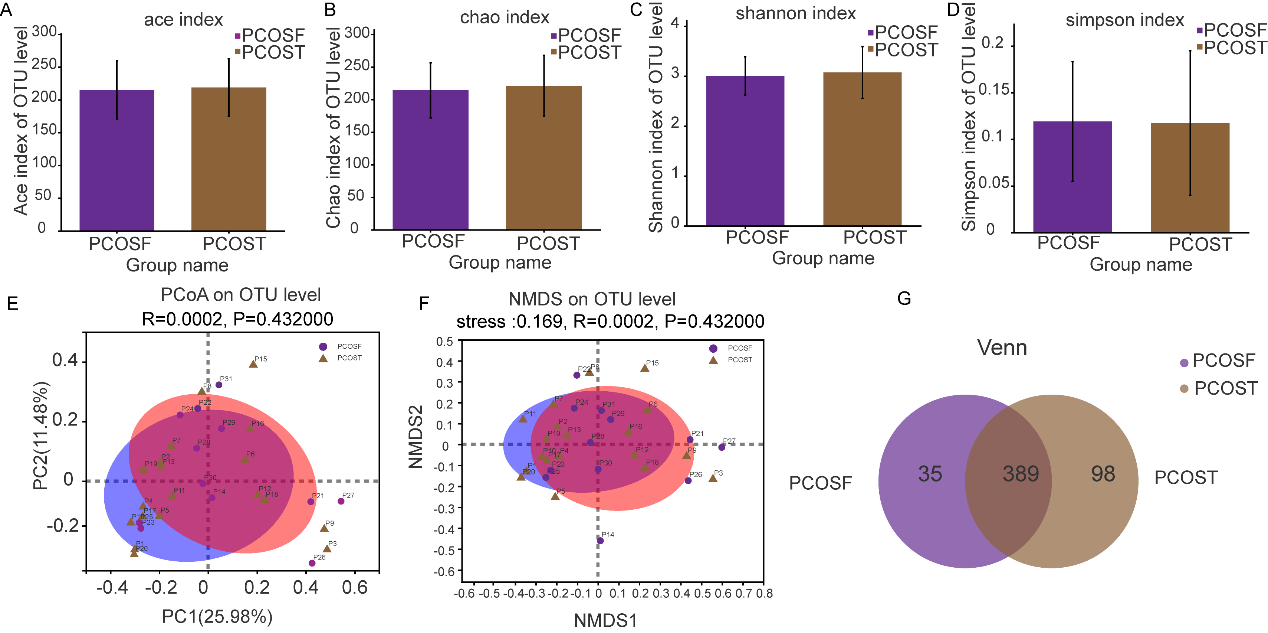
Figure 2**. (A, B, C, and D) Comparison of gut microbial α diversity index of PCOS patients with different body types. (E and F) PCoA and NMDS based on OTUs distribution showed β diversity differences between PCOS patients of different body sizes. (G) A Venn diagram shows the number of unique and common OTUs between the two groups. OTUs, Operational Taxonomic Units; PCOSF, PCOS patients with BMI≥24kg/m2 (N=12); PCOST, PCOS patients with BMI<24 kg/m2 (N=19). PCOSF, PCOS patients with BMI≥24kg/m2 (N=12); PCOST, PCOS patients with BMI<24 kg/m2 (N=19).

**Supplementary
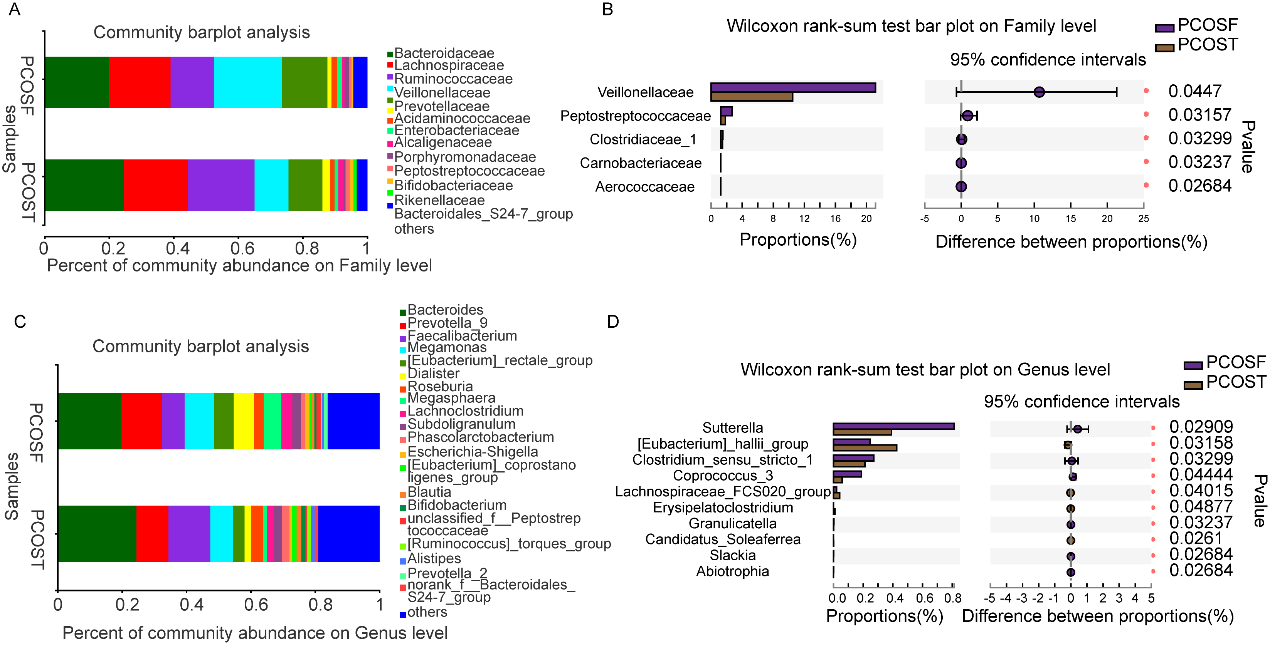
Figure 3**. (A, B, C and D) Composition and differences of the gut microbiome of PCOS with different body types at family and genus level. The difference of intestinal flora between the two groups was compared by STAMP analysis. PCOSF, PCOS patients with BMI≥24kg/m2 (N=12); PCOST, PCOS patients with BMI<24kg/m2 (N=19). *, P≤0.05; **, P≤0.01; ***, P≤0.001.


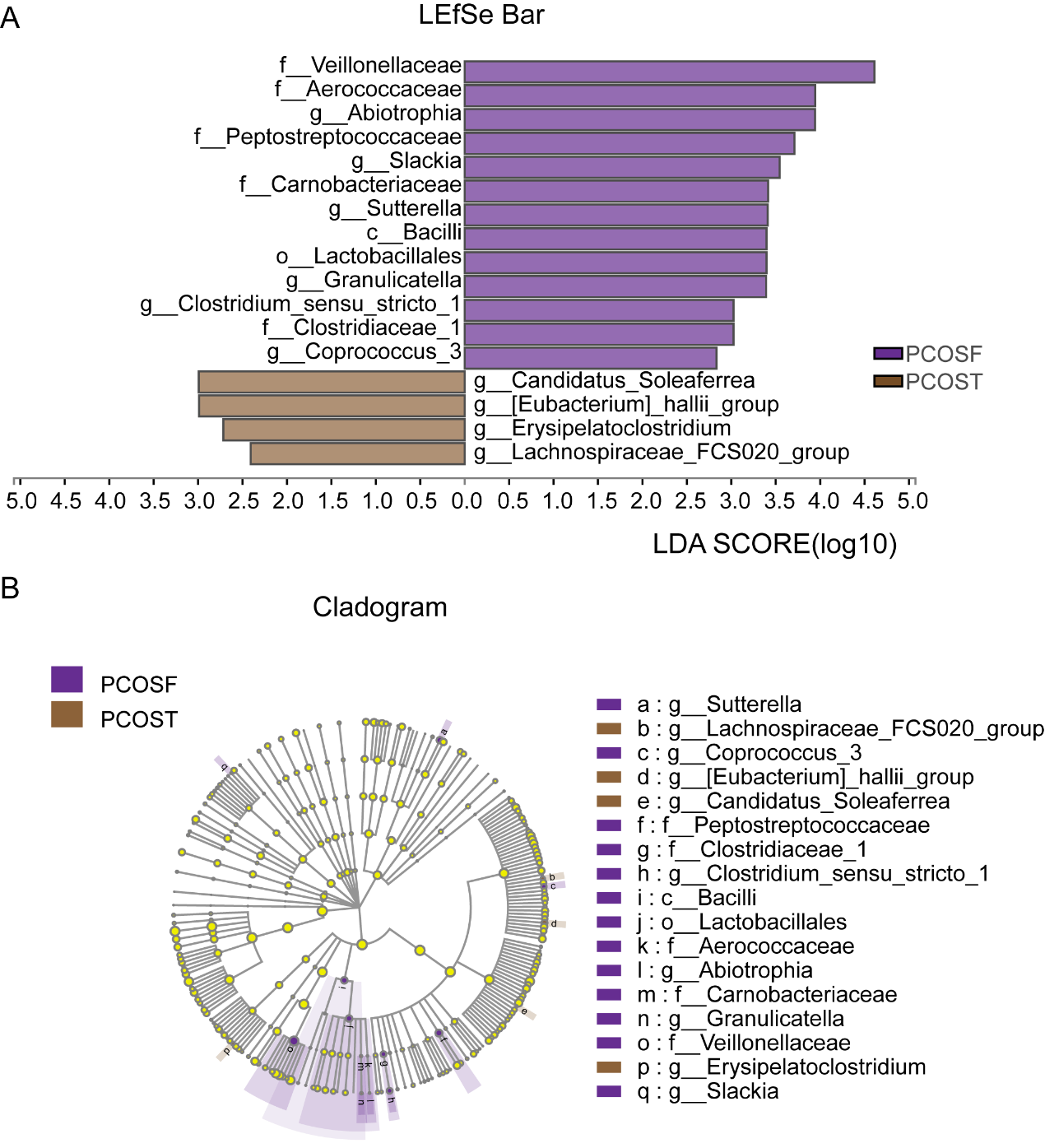
**Supplementary** **Figure 4**. (A) LDA branch histogram of dominant gut flora in PCOS with different body types (LDA score (log10)>2, P<0.05). (B) Phylogenetic profiles of specific bacterial groups and dominant flora between the two groups of gut microbiota. PCOSF, PCOS patients with BMI≥24kg/m2 (N=12); PCOST, PCOS patients with BMI<24 kg/m2 (N=19); LDA, linear discriminant analysis**.**
